# Editorial: Physical stimulus- performance-adaptation: understanding the physiological relationship

**DOI:** 10.3389/fphys.2023.1219034

**Published:** 2023-06-08

**Authors:** Gianpiero Greco, Antonella Muscella, Georgian Badicu, Francesco Fischetti

**Affiliations:** ^1^ Department of Translational Biomedicine and Neuroscience (DiBraiN), University of Study of Bari, Bari, Italy; ^2^ Department of Biological and Environmental Science and Technologies, University of Salento, Lecce, Italy; ^3^ Department of Physical Education and Special Motricity, Faculty of Physical Education and Mountain Sports, Transilvania University of Braşov, Braşov, Romania

**Keywords:** exercise, sport, performance, conditioning, adaptation

Performance can be defined as the accomplishment of goals by meeting or exceeding predefined standards (Portenga et al., 2017). Performance can be affected by stress (Bali, 2015), somatic maturation (Campa et al., 2019; Toselli et al., 2021) and training load as exposure and dose (Impellizzeri et al., 2023). Furthermore, if an individual’s recovery status (i.e., their biopsychosocial balance) is disturbed by external or internal factors, fatigue develops as a condition of increased tiredness due to physical and mental effort, reducing performance. Only through recovery can fatigue be compensated, thus restoring balance on a physiological and psychological level (Halson, 2014).

This Research Topic aimed to collect and disseminate the most current research examining on how stress, maturation, training load, and recovery tend to impact the performance and health of athletes at all ages. Since sport science research is still scarce on this Research Topic, scholars have provided an important contribution by investigating this stimulus-performance-adaptation relationship in different contexts and environments, with the aim of expanding our knowledge and the implications for practice.

Liu et al. tried to develop a strategy to improve the functionality of professional firefighters and help their work. Through a pilot, randomized, and controlled study, they investigated the effects of a 12-week complex training program (i.e., resistance training + plyometrics training or explosive exercises) on the strength, power, and rescue performance of professional firefighters. This pilot study demonstrated the effectiveness of complex training in inducing significantly greater improvements in the fire fighters’ strength and power compared to resistance training, thereby improving the firefighters’ ability to perform work-related tasks. In addition, the study provides tactical conditioning professionals with further suggestions for appropriately developing daily routine training programs. This study strengthens the previous findings of Fischetti et al. (2019) where complex training referred to as multilateral has improved the physical performance of law enforcement.

Jiang and Xu aimed to investigate the young basketball players’ lower limb explosive power training. Specifically, they explored the effects of the chain squat training with different chain load ratio (0, 10%, 20% and 30%) on the explosive power of the lower limbs of adolescent male basketball players. The findings showed that chain squat training with more chain load has better training effects on lower limb explosive strength and maximum strength, based on the improvement in 1RM squat and jumping performance. Besides, compared with traditional squat training, chain squat training with more chain load might not help to develop better velocity adaptation at a higher range of movement.

In their study, Patti et al. (2022a) had the purpose to evaluate the effects of an experimental short-time warm-up consisting of a small number of intermittent high-intensity sprints on explosive muscle strength performance in soccer players and to identify recovery times after performing the sprints. The results highlighted that a short-time high-intensity warm-up composed of intermittent sprints appears to be a simple, quick, and efficient activity to improve soccer players’ performance. These results reinforce the findings of the study by Patti et al. on elite female futsal athletes.

Since there is a paucity of data on physiological heart adaptation in elite-level judo female athletes, Milovančev et al. aimed to assess left ventricular morphology and function compared to non-athletes. The results showed that highly trained elite female judoka athletes exhibit significant changes in left heart morphology due to vigorous training compared to non-athletes. These findings suggest that the heart of female judoka athletes follows a pattern of chamber dilation rather than left ventricular wall hypertrophy.

Through a preliminary randomized controlled trial, García et al. aimed to describe and compare the recovery status of basketball players that underwent the NESA neuromodulation treatment and a placebo group in weeks with one and two matches. The results suggested that players who underwent the ESA neuromodulation treatment tended to improve their sleep quality even though their biochemical markers and wellness status remained like the placebo group. In addition, no significant differences were found between weeks with one or two games, which could help basketball professionals establish that a congested calendar would not seem to affect recovery status adversely.

Through a systematic review and meta-analysis, Hu et al. aimed to evaluate the effectiveness of transcranial alternating current stimulation on motor performance and learning in healthy individuals. The results showed that transcranial alternating current stimulation effectively improves motor performance and motor learning in healthy individuals, which indicates that it may be a potential therapeutic tool to improve motor behavioral outcomes.

Since it is unclear whether the different dynamic balance and power demands produced by the lower limb can be observed in sport-specific differences between gymnasts of various modalities, Kyselovičová et al. aimed to compare variables of dynamic balance and isokinetic leg muscle strength in rhythmic, artistic, and aerobic gymnasts. The results showed a significant association between the symmetry of the dominant limb and the isokinetic extension strength of the dominant limb; therefore, it can be concluded that both muscle strength and speed contribute to dynamic balance in adolescent gymnasts.

Finally, Xiao et al., through a systematic review and meta-analysis, have examined the effect of cold-water immersion on post-exercise fatigue recovery after high-intensity exercise. The results showed that cold water immersion could effectively reduce muscle soreness and speed up recovery from fatigue.

In conclusion, this Research Topic provided multidisciplinary investigations on the physiological relationships between stimulus-performance-adaptation in different contexts and environments. We have observed how training stress, biological maturation, training load and recovery tend to influence the performance and health of tactical populations and athletes of different sports and ages.

Overall, some researchers have encouraged future research to address open Research Topic about this topic. From previous studies, the need to develop more appropriate training programs for firefighters in their daily routine has emerged. Further studies are needed on warm-up as it is a complex Research Topic, as changes in volume and intensity and recovery could negatively affect performance. Further studies are needed to strengthen knowledge of the specific adaptations of cardiac structure and function in judokas, which play a crucial role in classifying cardiac remodeling and distinguishing between pathophysiology and sport-specific individual adaptations. Finally, more research evidence is needed to verify the efficacy of transcranial alternating current stimulation on motor learning.

## References

[B1] Bali A. (2015). Psychological factors affecting sports performance. Int. J. Phys. Educ. Sports Health 1 (6), 92–95.

[B2] Campa F. Silva A. M. Iannuzzi V. Mascherini G. Benedetti L. Toselli S. (2019). The role of somatic maturation on bioimpedance patterns and body composition in male elite youth soccer players. Int. J. Environ. Res. Public Health 16 (23), 4711. 10.3390/ijerph16234711 31779215 PMC6926995

[B3] Fischetti F. Cataldi S. Latino F. Greco G. (2019). Effectiveness of multilateral training didactic method on physical and mental wellbeing in law enforcement. J. Hum. Sport Exerc. 14 (4), S900–S909. 10.14198/jhse.2019.14.Proc4.53

[B4] Halson S. L. (2014). Monitoring training load to understand fatigue in athletes. Sports Med. 44 (2), 139–147. 10.1007/s40279-014-0253-z PMC4213373 25200666

[B5] Impellizzeri F. M. Shrier I. McLaren S. J. Coutts A. J. McCall A. Slattery K. (2023). Understanding training load as exposure and dose. Sports Med., 1–13. 10.1007/s40279-023-01833-0 PMC10432367 37022589

[B6] Patti A. Giustino V. Cataldi S. Stoppa V. Ferrando F. Marvulli R. (2022a). Effects of 5-week of FIFA 11+ warm-up program on explosive strength, speed, and perception of physical exertion in elite female futsal athletes. Sports 10, 100. 10.3390/sports10070100 35878111 PMC9322867

[B7] Portenga S. T. Aoyagi M. W. Cohen A. B. (2017). Helping to build a profession: A working definition of sport and performance psychology. J. Sport Psychol. Action 8 (1), 47–59. 10.1080/21520704.2016.1227413

[B8] Toselli S. Campa F. Maietta Latessa P. Greco G. Loi A. Grigoletto A. (2021). Differences in maturity and anthropometric and morphological characteristics among young male basketball and soccer players and non-players. Int. J. Environ. Res. Public Health 18 (8), 3902. 10.3390/ijerph18083902 33917743 PMC8068181

